# Endosymbionts that threaten commercially raised and wild bumble bees (*Bombus* spp.)

**DOI:** 10.26786/1920-7603(2023)713

**Published:** 2023-02-07

**Authors:** Laura L. Figueroa, Ben M. Sadd, Amber D. Tripodi, James P. Strange, Sheila R. Colla, Laurie Davies Adams, Michelle A. Duennes, Elaine C. Evans, David M. Lehmann, Heather Moylett, Leif Richardson, James W. Smith, Tamara A. Smith, Edward M. Spevak, David W. Inouye

**Affiliations:** 1 Department of Environmental Conservation, University of Massachusetts, Amherst, Amherst, MA, 01003, USA; 2 Department of Entomology, Cornell University, Ithaca, NY, 14850, USA; 3 School of Biological Sciences, Illinois State University, Normal, IL 61790, USA; 4 Unaffiliated, Raleigh, North Carolina, 27604, USA; 5 Department of Entomology, The Ohio State University, Columbus, OH 43214, USA; 6 Faculty of Environmental and Urban Change, York University, Toronto, ON, Canada; 7 Pollinator Partnership, 600 Montgomery, Suite 440, San Francisco, CA 94111, USA; 8 Department of Biology, Saint Vincent College, Latrobe, PA 15650, USA; 9 Department of Entomology, University of Minnesota, Saint Paul, MN 55108 USA; 10 Center for Public Health and Environmental Assessment (CPHEA), Health and Environmental Effects Assessment Division, Integrated Health Assessment Branch, US - Environmental Protection Agency, Research Triangle Park, NC, 27711, USA; 11 Unaffiliated, Raleigh, North Carolina, 27604, USA; 12 The Xerces Society for Invertebrate Conservation, 628 NE Broadway, Suite 20, Portland, OR 97232-1324, USA; 13 Retired USDA-Animal and Plant Health Inspection Service, Raleigh, NC 27526, USA; 14 US Fish & Wildlife Service, Minnesota/Wisconsin Ecological Services Field Office, 4101 American Boulevard East, Bloomington, MN 55425, USA; 15 Center for Native Pollinator Conservation, Saint Louis Zoo, One Government Drive, St. Louis, MO 63110, USA; 16 Department of Biology, University of Maryland, College Park, MD 20742, and Rocky Mountain Biological Laboratory, PO Box 519, Crested Butte, CO 81224, USA

**Keywords:** *Bombus*, bumble bee, symbionts, parasites, pathogens

## Abstract

Bumble bees (*Bombus* spp.) are important pollinators for both wild and agriculturally managed plants. We give an overview of what is known about the diverse community of internal potentially deleterious bumble bee symbionts, including viruses, bacteria, protozoans, fungi, and nematodes, as well as methods for their detection, quantification, and control. We also provide information on assessment of risk for select bumble bee symbionts and highlight key knowledge gaps. This information is crucial for ongoing efforts to establish parasite- conscious programs for future commerce in bumble bees for crop pollination, and to mitigate the problems with pathogen spillover to wild populations.

## Introduction

Bumble bees (*Bombus* species) are widespread globally, but most prevalent and diverse at high altitudes and high latitudes, where they can constitute a large proportion of the pollinator fauna. There is reason for concern about their status, as declines have been reported around the world (Arbetman et al. 2017; Graves et al. 2020; Soroye et al. 2020; Van Dooren 2019), and a North American species has been listed as endangered and hasn’t been seen since 2006 (https://biologicaldiversity.org/w/news/press-releases/elusive-pacific-northwest-bumblebee-listed-as-endangered-2021-08-23/; accessed 18 January 2023). Bumble bees are important pollinators for both wild and cultivated plants, and there is now a thriving trade in commercial colonies, shipped to many parts of the world, for pollination of crops such as tomatoes, blueberries, and raspberries, both in greenhouses and open fields (Velthuis & van Doorn 2006). The loosely regulated trade of commercial bumble bees has led to the introduction of a variety of endoparasites, with possible spillover to conspecifics and other species in the wild bumble bee community, or to native species when the commercial species are non-native (Colla et al. 2006; Graystock et al. 2013b). We are still discovering the extent and frequency of parasite spread to wild communities through commerce, and the consequences of these introductions for native bees. As a prelude to potential regulation, such as a “clean stock” mandate that bees be certified as parasite-free before being shipped (Strange et al. 2023), it is important to know the diversity of known bumble bee endosymbionts, their impacts on hosts (when known), and potential mechanisms for preventing future introductions. Here we review the literature and describe the diversity, pathology, and detection methods for bumble bee viruses, bacteria, protozoans, fungi, and nematodes, as well as associated knowledge gaps.

### 1) Symbionts potentially deleterious to bumble bees

In this section, we address some of the most important, most commonly encountered, and best-known potentially deleterious internal symbionts of bumble bees (i.e., endosymbionts), particularly those that are of interest in captive rearing environments. This is far from a complete list (See supplementary Symbiont List in Appendix I) but interested readers who wish to learn about some of the more obscure organisms associated with bumble bees are encouraged to seek the works on parasitism (Beaurepaire et al. 2020; de Miranda et al. 2013; Macfarlane et al. 1995; Schmid-Hempel 1999) and bumble bee natural history (Alford 1975; Goulson 2010). Additionally, we use the term “parasite” broadly to refer to organisms of all taxa, including viruses, that sustain themselves at the expense of their hosts and have the potential to cause harm to their hosts, a definition which, for our purposes, also encompasses the term “pathogen”. Here we focus on known bumble bee endosymbionts; a review of known bumble bee ectosymbionts can be found in Evans et al. (2023). Recommendations for implementing a clean stock program to detect and prevent the spread of parasites of concern in commercial rearing facilities that pose a threat to wild bees can be found in Strange et al. (2023).

#### Viruses

To date, all of the named viruses detected in bumble bees have previously been reported from honey bees. There are approximately 60 honey bee viruses currently known, although next-generation sequencing technologies are allowing for the exploratory discovery of additional viruses of managed honey bees and wild bees (Beaurepaire et al. 2020; de Miranda et al. 2013; Remnant et al. 2017; Schoonvaere et al. 2016). A single virus, perhaps specific to bumble bees, was noted in three North American species in the 1980s (present in *B. pensylvanicus, B. impatiens*, and *B. fervidus*; absent in *B. bimaculatus* and *B. vagans*), but nothing is known about these “entomopoxvirus-like particles”, aside from their original description (Clark 1982). Most honey bee-associated viruses found in bumble bees are single-stranded, positive-strand RNA (ss-RNA) viruses. The structure of these ss-RNA viruses allows for the diagnosis of active replication through detection of the negative (replicating) strand. Although negative-strand detection has indicated that the so-called honey bee viruses do replicate within bumble bees (Fürst et al. 2014; Li et al. 2011; Radzevičiūtė et al. 2017), the effects of infection on individuals and colonies are largely unknown, and it is not clear whether presence of these viruses is maintained largely through spillover or whether substantial transmission occurs within the wild bee community (Manley et al. 2015). Many honey bee viruses persist within honey bee colonies as non-apparent, chronic infections that exhibit symptoms only when the colony is exposed to additional stressors or intracuticular exposure, such as seen with the strains transmitted by *Varroa* mites (McMenamin et al. 2016). Although these viruses are considered honey bee viruses, there is little known of their true host ranges or their ability to cause disease in non-*Apis* hosts (Tehel et al. 2016).

Deformed Wing Virus (DWV) is one of the most commonly detected honey bee viruses in both Europe and North America (Dolezal et al. 2016; McMahon et al. 2015). DWV is known to affect colonies negatively and can be transferred by feeding on infected pollen. Although infected individuals often eclose as adults with crippled wings, cryptic and asymptomatic infections are known, and other factors can deform the wings of bees during pupation, including infections of *Vairimorpha* (*Nosema*) *bombi* (Rutrecht & Brown 2009). The first detection of the virus in bumble bees was based on visual inspection of overt pathology. In a commercial rearing facility in Europe, about 10% of new *B. terrestris* queens exhibited characteristic crumpled wings upon eclosion, and these, as well as asymptomatic honey bees in a co-located apiary, were shown to be harboring DWV (Genersch et al. 2006). The host range of DWV might be quite broad, however, as replicating DWV has been found in a number of insect orders, including Blattodea and Dermaptera, as well as in *Varroa destructor*, a member of the class Arachnida and an ectoparasite of honey bees (Gisder & Genersch 2016; Manley et al. 2015). A recent study has documented the ongoing potential replacement of genotypes of DWV in honey bees (Paxton et al. 2022).

Using molecular means, DWV has been detected across a broad spectrum of wild bee hosts in many families. In the United Kingdom, asymptomatic cases of DWV have been detected in wild, flying individuals of *B. terrestris* and *B. pascuorum*, as well as in the wasp *Vespula vulgaris* (Evison et al. 2012). Prevalence of DWV is often quite high in some of the insect populations surveyed, (*e.g., Apis mellifera* (100%); *B. terrestris* (29%), and the wasp *V. vulgaris* (30%)), although other species of bumble bees surveyed at these same sites were free of DWV (Evison et al. 2012). DWV has also been detected in North American bumble bee species, including field-collected *B. ternarius* and *B. vagans*, wild and lab-reared *B. huntii*, and commercially sourced *B. impatiens* (Levitt et al. 2013; Li et al. 2011; Sachman-Ruiz et al. 2015; Singh et al. 2010). The virus has also been observed in bumble bees from commercially sourced colonies in Europe (Evison et al. 2012; Graystock et al. 2013b). In North America, active replication of DWV has been observed in *B. huntii, B. impatiens*, and *B. vagans* (Levitt et al. 2013; Li et al. 2011). There were no measurable differences between quantified levels of virus in wild bees and wild-caught honey bees in a study in the United States, although wild-caught honey bees had much higher levels in a quantification study in the United Kingdom (Dolezal et al. 2016; McMahon et al. 2015). In a survey of *B. atratus* in Colombia, 100% of the bees from seven nests screened for parasites had DWV (Gamboa et al. 2015) and both native and introduced species of *Bombus* in Argentina were found to have a variety of viral pathogens (Arismendi et al. 2021).

Few experiments have addressed the incidence of disease in DWV-infected bumble bees, but DWV has been shown to increase mortality in experimentally infected individuals both alone and with co-infection with the protozoan *Apicystis bombi* (Fürst et al. 2014; Graystock et al. 2016). Although a laboratory study considering the efficacy of proposed natural transmission routes suggested that transmission in the wild may be limited (Gusachenko et al. 2020; Streicher et al. 2023), research has demonstrated spillover from honey bees to bumble bees (Tehel et al. 2022) and a potential introduction with non-native bumble bees (Arbetman et al. 2013). The closely related *Varroa destructor* viruses (VDVs) and kakugo virus (KV) are considered by some to be variants of a DWV species complex (McMahon et al. 2015). Alger et al. (2019) examined spillover of honey bee viruses to wild bumble bees and found DWV and Black Queen Cell Virus (BQCV) to be higher in bumble bees foraging in areas where apiaries were found. Additionally, they confirmed the presence of these viruses on flowers near apiaries, which indicates the potential for spread of bee viruses due to shared flower use in agricultural landscapes where managed bees are most commonly used.

Acute Bee Paralysis Virus (ABPV), Kashmir Bee Virus (KBV), and Israeli Acute Paralysis Virus (IAPV) are closely related and considered strains of the same virus complex (AKI-complex) (Gisder et al. 2009; McMahon et al. 2015). ABPV was the first honey bee virus to be detected in bumble bee hosts, and all bumble bee species tested are susceptible to experimental infection and show classic symptoms, although its occurrence in natural populations and effect on bumble bee health through natural infection routes are unknown (Bailey & Gibbs 1964). In honey bees, ABPV causes trembling, loss of motor control, and eventual death within a few days of infection (Bailey & Gibbs 1964). ABPV is systemic but found in high concentrations within the salivary glands of honey bees and can be transmitted through pollen, honey, and trophallaxis (Bailey & Gibbs 1964; Benjeddou et al. 2001). The virus is shed in large quantities in feces and remains infectious for months (Bailey & Gibbs 1964).

A recent survey in the United Kingdom found ABPV to be the most common virus detected in bumble bees, and that ABPV was more common in bumble bees than in honey bees collected from the same sites (McMahon et al. 2015). Commercial colonies of *B. impatiens* in Mexico also tested positive for ABPV (Sachman-Ruiz et al. 2015). ABPV was detected in wild *B. atratus* in Colombia, though in lower prevalence than other viruses in the screening (Gamboa et al. 2015). Although KBV has been reported from bumble bees in North America and New Zealand, these records are vague and do not include which species were infected (Singh et al. 2010; Ward et al. 2007). However, one colony of commercial *B. impatiens* tested positive for KBV in Mexico (Sachman-Ruiz et al. 2015). KBV is detectable in feces, suggesting this may be a relevant infection route for foraging bees sharing floral resources (Hung 2000).

In addition to detection within *Bombus* spp., there is some information on the transmission and virulence of viruses in the AKI-complex for *Bombus*. IAPV causes shivering, paralysis, and death in infected honey bees, with increased mortality in the presence of *Varroa* (Gisder et al. 2009; Palacios et al. 2008). IAPV has been detected in commercially reared *B. impatiens*, and cross-infectivity studies suggest that transmission between honey bees and bumble bees can occur through shared food sources (Sachman-Ruiz et al. 2015; Singh et al. 2010). The route of infection may be very important to the virulence of this virus complex. Orally administered IAPV and KBV did not induce mortality in infected *B. terrestris* individuals, but KBV-infected microcolonies suffered slower colony establishment and lower offspring production, with the latter also seen for IAPV (Meeus et al. 2014). A subsequent study has shown that oral administration can result in acute infections with associated virulence, but at much higher doses (Wang et al. 2018). Another study showed that injections of as few as 20 particles of IAPV into *B. terrestris* caused rapid mortality, with all experimental bees dead after only eight days; in contrast, bees injected with as many as 20,000 particles of another, unrelated virus, Slow Bee Paralysis Virus (SBPV), showed no increase in mortality over control bees (Niu et al. 2016). Yet, SBPV virulence can be condition-dependent, with even orally administered SBPV increasing *B. terrestris* mortality under nutritional limitation (Manley et al. 2015). SBPV has also been detected in bumble bees from the United Kingdom, at a slightly, but non-significantly, higher prevalence than honey bees, whereas IAPV was not detected in either host (McMahon et al. 2015).

In honey bees, Chronic Bee Paralysis Virus (CBPV) is recognizable by the presence of congregations of trembling bees at the hive entrance, yet infections rarely impact colonies unless other stressors, such as overcrowding or nutritional stress, are also present (Allen & Ball 1996). Replicating CBPV has been detected in non-*Apis* organisms, including the mite *Varroa destructor*, and the ant *Camponotus vagus*, which opportunistically feeds on dead honey bees, suggesting a wider host range for this virus than is currently documented (Celle et al. 2008). CBPV was tied with ABPV for the most common virus detected in commercial colonies of *B. impatiens* in Mexico (Sachman-Ruiz et al. 2015), and it has also been detected in native bumble bees in Argentina (Fernandez de Landa et al. 2020) and Colombia (Gamboa et al. 2015). Cloudy Wing Virus (CWV, initially described as CW Particle) is a similar, but likely unrelated virus (Bailey et al. 1980). There are few data about the pathology of this virus, even in honey bees. It appears to exist primarily as an asymptomatic infection in honey bees, although under some circumstances, it may cause rapid mortality (Bailey et al. 1980; Carreck et al. 2010). In Korea, the virus has been detected in captive, field-deployed colonies of *B. terrestris* and *B. ignitus*, and may have been an agent of mortality when present in combination with other viruses, such as KBV and Sacbrood virus (SBV) (Choi et al. 2010).

Black Queen Cell Virus (BQCV) is one of the most common honey bee viruses and has been detected in multiple hymenopteran hosts, including ants, wasps, and bees including miner (Andrenidae), sweat (Halictidae), carpenter (Xylocopa; Apidae), leaf-cutting (Megachilidae) and bumble (Bombus; Apidae) bees (Levitt et al. 2013; Peng et al. 2011; Ravoet et al. 2014; Singh et al. 2010; Zhang et al. 2012). The distribution of the virus is largely unknown, but, due to its prevalence in honey bees (*e.g*., 98.5% of sampled honey bees in Pennsylvania (Singh et al. 2010)), it is expected to be widespread. Bumble bees from commercial facilities have been recorded harboring the virus in the United States (Singh et al. 2010), Mexico (Sachman-Ruiz et al. 2015), and Argentina (Reynaldi et al. 2013), as have both laboratory-reared and field-caught *B. huntii* in Utah, United States (Peng et al. 2011), and wild bumble bees in Colombia (Gamboa et al. 2015). Replicating BQCV in bumble bees has also been detected in multiple sites across Europe (Radzevičiūtė et al. 2017). Field surveys show that BQCV is common in both honey bees and bumble bees in the United Kingdom (McMahon et al. 2015), but a study in Iowa (United States) detected very few bumble bees with the virus, in spite of high prevalence in apiaries (Dolezal et al. 2016). BQCV has been detected in pollen loads harvested from honey bee workers (Singh et al. 2010), and in wild bumble bees foraging near apiaries (Alger et al. 2019; McNeil et al. 2020). BQCV replicates in the tissues of the midgut of *B. huntii* and is distributed throughout the body, yet infected individuals show no overt symptoms (Peng et al. 2011). In honey bees, infection by BQCV is more detrimental to larvae, with adults only suffering from infection when coinfected with the microsporidian *Vairimorpha apis* (Ball & Bailey 1999). If such age-specific effects of BQCV infection are also present in bumble bees, it may be difficult to assess the presence and effects of BQCV infections, although Salvarrey et al. (2021) were able to detect BQCV in over 90% of *B. pauloensis* workers in the wild.

Sacbrood virus (SBV) is a disease that causes mortality in honey bee larvae. Infected individuals cannot molt and eventually die, leaving distinctive carcasses full of virus-laden ecdysial fluid that are usually removed from the colony by vigilant workers (Bailey 1975). Although the effect of SBV infection on bumble bees is unknown, it has been detected in non-*Apis* hosts on three continents, including in *B. ternarius, B. vagans, B. atratus, Andrena* spp., and the paper wasp *Polistes metricus* (Ravoet et al. 2014; Reynaldi et al. 2013; Singh et al. 2010). The virus can also be detected in pollen collected by foraging honey bees (Singh et al. 2010), suggesting a possible transmission route to captive-reared bumble bees. In a sample of 33 wild bumble bee individuals from Iowa, SBV was the most commonly detected virus of five tested for, with 52% testing positive for SBV (Dolezal et al. 2016). However, there have not been any studies that have tested for replicating strands of SBV or examined the impacts of SBV infection on bumble bees, so the impact of this virus is unknown (Gisder et al. 2009).

Bumble bees have been surveyed for only a few honey bee viruses, yet these pathogens appear common among many species and across a wide geographic range. There will likely be more honey bee viruses detected in bumble bees, given that others, such as *Apis mellifera* Filamentous Virus (AmFV), have been detected in more distantly related solitary bees, such as *Andrena vaga, A. ventralis, Osmia bicornis* and *O. cornuta* (Ravoet et al. 2014). AmFV was recently discovered in native *Bombus* in the Andes (Plischuk et al. 2021). Unraveling the infection dynamics, routes of transmission, and distinct physiological and colony-level effects of these viruses on bumble bee hosts will be necessary to determine the impacts of honey bee viruses on bumble bee hosts (Tehel et al. 2016).

#### Bacteria

Little is known about bacterial diseases in bumble bees, but early reports speculated that pathogenic bacteria were responsible for some larval mortality (Frison 1926). More recently there has been a focus on the beneficial effects of core bacteria associated with the gut of Apid bees (Kwong & Moran 2016), and how these microbes may aid in resistance against parasite infection (Koch & Schmid-Hempel 2011a; Koch & Schmid-Hempel 2011b; Mockler et al. 2018). While bacterial diseases of honey bees such as American foulbrood (*Paenibacillus larvae*) and European foulbrood (*Melissococcus plutonius*) can be devastating, there are few homologous reports of bacterial infections in bumble bees (Fünfhaus et al. 2018). Many bacteria that have been found in bumble bees to date appear to be largely either commensal or beneficial, though further work is warranted on this topic. Bacteria that have been identified from bumble bees include *Bacillus cereus, B. pumilus, Brevibacillus laterosporus, Burkholderia cepacia, Enterobacter* (formerly *Aerobacter*) *cloacae, Lysinibacillus* (as *Bacillus*) *fusiformis, Paenibacillus glucanolyticus*, *Spiroplasma apis* and *S. melliferum* (Ahmed et al. 2007; Macfarlane et al. 1995; Marche et al. 2016; Meeus et al. 2012; Přidal 2001; 2002; Přidal et al. 1997; Schmid-Hempel 1999).

*Spiroplasma melliferum* and *S. apis* are pathogenic bacteria that are associated with May disease in honey bees and both are known to cause mortality (Clark et al. 1985; Meeus et al. 2012). Although both are normally associated with honey bees, they have been detected on the surface of flowers and within the hemolymph and guts of numerous flower-visiting insects, including *B. impatiens, B. pensylvanicus, B. pascuorum, B. pratorum*, and *B. atratus*, and the leaf-cutting bees *Osmia cornifrons* and *O. bicornis* (Clark et al. 1985; Gamboa et al. 2015; Meeus et al. 2012; Ravoet et al. 2014). The presence of high levels of bacteria, like *Spiroplasma spp*., in bumble bee guts may indicate their potential as a pathogen in bumble bees (Clark et al. 1985), but this has not been verified. In honey bee queens, *E. cloacae* causes B-melanosis, a disease of the ovaries that sterilizes the queen (Fyg 1964), but its effect in bumble bees is unrecorded (Schmid-Hempel 1999). Bumble bees have rarely been screened for the presence of *Wolbachia*, but there are records of this bacterium being detected in European bumble bee species (Evison et al. 2012; Gerth et al. 2015). The effects of *Wolbachia* on hosts are complex (Werren et al. 2008); it is predominantly vertically transmitted and not always pathogenic. To date, we have no knowledge of the kind of association this bacterium has with bumble bees. Research on impacts of bacterial infections and microbiome studies are urgently needed to understand better how bacteria should be managed in a clean stock program.

#### Protozoans

The trypanosomatid *Crithidia bombi* is an intestinal parasite found in species throughout the genus *Bombus*, with a worldwide distribution (Schmid-Hempel & Tognazzo 2010). The distribution of this parasite within *Bombus* remains relatively poorly studied and most information on its pathology comes from *B. terrestris* and *B. impatiens*. A close relative, *C. expoeki*, was described from *Bombus* samples collected in both Europe and North America and is assumed to be a similar pathogen (Schmid-Hempel & Tognazzo 2010). In a survey throughout the United States, *C. bombi* was far more common than *C. expoeki* and co-occurred in the same hosts (Tripodi et al. 2018). Similarly, genetic data indicate another undescribed species, nicknamed “*C. mexicana*”, that was detected in bumble bee samples from southern Mexico (Gallot-Lavallée et al. 2016), and additional undescribed trypanosomatids in the United States (Tripodi et al. 2018). In the US, *C. bombi* prevalence is highly variable, but can be quite high, for example ranging from 0 – 82 % in Massachusetts (Gillespie 2010). An extensive survey of bumble bees in the USA found *Crithidia* to be widespread, yet at low prevalence across species at the sites sampled (Cordes et al. 2012), however another study found regional variation in infection rates (Tripodi et al. 2018). In addition to in *Bombus, C. bombi* has been detected in the non-Apidae hosts *Andrena vaga* and *Osmia bicornis* in Europe (Ravoet et al. 2014), including experimental evidence for active replication in *O. lignaria* and *M. rotundata* (Figueroa et al. 2021; Ngor et al. 2020), though nearly nothing is known about the pathogenicity of *Crithidia* in non-*Bombus* hosts (Figueroa et al. 2021). The honey bee trypanosomatid parasite *Lotmaria passim* has been detected molecularly in bumble bees from the United States, but may not be a true parasite of bumble bees (Tripodi et al. 2018). *Lotmaria passim* has also been found in wild bumble bees in the Andes mountains (Plischuk et al. 2021).

*Crithidia* parasites are flagellated and are found in the gut lumen of the host bee, anchoring to the ileum epithelium with their flagellum (Koch et al. 2019). Infection in bumble bees can impair the foraging abilities of infected workers (Gegear et al. 2005; Otterstatter et al. 2005), reduce queen hibernation survival (Fauser et al. 2017), and reduce colony founding success (Brown et al. 2003). Although acute mortality is rarely observed (Brown et al. 2003), under conditions of nutritional stress, infected workers are 50% more likely to succumb to infections than their well-fed counterparts (Brown et al. 2000). In general, the outcomes of infection are considered to be context- and condition-dependent (Sadd & Barribeau 2013).

*Crithidia* is shed in the feces and can be transmitted through feeding. Experimental evidence shows that bumble bees can contract *C. bombi* infections while feeding on flowers that have been previously visited by infected bees (Adler et al. 2020; Durrer & Schmid-Hempel 1994). Transmission dynamics on flowers vary by plant species and environmental conditions, with deposition and acquisition for foraging *B. impatiens* varying by flower parts, and exposure to UV radiation significantly reducing pathogen survival on flowers (Figueroa et al. 2019). Moreover, differences among plant species in transmission potential for individual *B. impatiens* workers (Adler et al. 2018) can affect colony-level infection patterns (Adler et al. 2020), highlighting the role of flowers in mediating transmission and prevalence in this bumble bee species. However, there is very limited understanding of *C. bombi* transmission patterns via flowers beyond *B. impatiens* and *B. terrestris* (Ruiz-González et al. 2012). Bees from commercial rearing facilities have tested positive for this parasite upon delivery (Gegear et al. 2005; Graystock et al. 2013b; Murray et al. 2013; Otterstatter et al. 2005). Higher infection levels were found in wild bumble bees near greenhouses that had deployed commercial bumble bees than in wild populations far removed from such sites, lending support to the “spillover hypothesis” (Colla et al. 2006; Graystock et al. 2014).

The neogregarine, *Apicystis bombi*, is a widely distributed parasite of multiple bumble bee species (Lipa & Triggiani 1996). In bumble bees, although there are few experimental assessments of virulence, the parasite can have severe effects. *Apicystis bombi* decimates the fat body of infected individuals, and field-collected infected spring queens of European species die before founding colonies (Jones & Brown 2014; Rutrecht & Brown 2008). Commercially sourced colonies of *B. terrestris* were found to harbor this parasite, suggesting a real danger of pathogen spillover of this organism from captive to wild populations (Graystock et al. 2013b). Unlike *Crithidia, Apicystis* was not associated with greenhouse sites in a Canadian study, although a study in the United Kingdom did see higher prevalence of both parasites near greenhouse sites (Colla et al. 2006; Graystock et al. 2014). Population genetics of *A. bombi* from Argentina, Colombia, Mexico, and Europe also suggest that *A. bombi* in Argentina may have originated from the recent importation of non-native *B. terrestris* from Europe to Chile as commercial pollinators (Aizen et al. 2019; Maharramov et al. 2013). However, *B. terrestris* has not been documented in Colombia, thus the high prevalence of *A. bombi* in South America might be due to more complex factors (Gamboa et al. 2015). Feeding experiments show that *A. mellifera* are susceptible to *A. bombi* infections, and this parasite has been infrequently reported from *A. mellifera* in Europe, Japan, and South America (Graystock et al. 2013a; Lipa & Triggiani 1996; Morimoto et al. 2013; Plischuk et al. 2011; Ravoet et al. 2014; Schulz et al. 2019). Additionally, it has been detected in European specimens of *Andrena vaga, A. ventralis, Heriades truncorum, Osmia bicornis*, and *O. cornuta* (Ravoet et al. 2014). *Apicystis cryptica* was recently described from *B. pascuorum* from Belgium (Schoonvaere et al., 2020), but this species has not been reported from bees in the United States (Ivers et al. 2022).

#### Fungi

The microsporidian *Vairimorpha* (*Nosema*) *bombi* (Tokarev et al. 2020) has a cosmopolitan distribution (Cameron et al. 2016; Koch & Strange 2012; Li et al. 2011) and is found throughout the genus *Bombus*; however, evidence suggests that some species and/or subgenera are differentially infected (Cameron et al. 2011; Cordes et al. 2012). Furthermore, some declines of bumble species have been linked to presumed epizootic events involving *V*. *bombi*, including the recent declines of the North American subgenera *Bombus sensu stricto* and *Thoracobombus* (Cameron et al. 2011; Malfi et al. 2014). However, while the incidence of *V. bombi* in North America has increased in recent times, there is currently no evidence to support the hypothesis that contemporary strains of the parasite were exotic or introduced from Europe (Cameron et al. 2016). *Vairimorpha bombi* has frequently been detected in commercially sourced colonies and greenhouse-associated wild populations, but the evidence for spillover remains inconsistent and inconclusive (Colla et al. 2006; Graystock et al. 2013b; Murray et al. 2013; Sachman-Ruiz et al. 2015; Whittington & Winston 2003).

Infections of *V. bombi* occur through the digestive tract, with spores usually concentrated in the Malpighian tubules, the tissues of the midgut and the fat body, although spores can also present in muscles, and the accessory glands, ovaries, accessory testes, and testes of reproductive adults (Larsson 2007; Otti & Schmid-Hempel 2007). Bumble bee colonies that are infected with *V. bombi* can suffer from a reduction in reproductive capacity (van Der Steen, 2008). Mortality is higher in infected males, and the survivors produce fewer viable sperm, while infected gynes exhibit swollen abdomens and are more hesitant to mate than their uninfected counterparts (Otti & Schmid-Hempel 2007). Infections of colonies early in the colony cycle lead to an absence of the production of sexuals (Otti & Schmid-Hempel 2008). However, other studies have found *V. bombi* to have no effect upon colony growth or reproductive output (Whittington & Winston 2003). Much of what is known about the pathology of *V. bombi* infections is from a limited number of species (*B. terrestris* and *B. lucorum*), and species may be differentially affected by the disease (Brown 2017). For example, although infected colonies of *B. lucorum* were less likely to produce gynes, when they were produced, they were fully functional and capable of mating, unlike the gynes produced in *B. terrestris* colonies (Rutrecht & Brown 2009). Recently, *B. impatiens* males were shown to have a high tolerance to experimentally established *V. bombi* infections (Calhoun et al. 2021).

Recent molecular screening of *V. bombi* in wild bee communities across old fields and wildflower strips in upstate NY (USA) found the pathogen to be virtually absent across two years of sampling (Figueroa et al. 2019; Graystock et al. 2020), highlighting that factors that contribute to differing prevalence rates are not sufficiently understood. Conversely, bumble bees in Argentina, Colombia, the United Kingdom, the USA, and Uruguay have regularly tested positive for *V. ceranae* (*Nosema ceranae*), with low/absent prevalence of *V. apis* (*Nosema apis*), (Arbulo et al. 2015; Figueroa et al. 2019; Fürst et al. 2014; Gamboa et al. 2015; Graystock et al. 2014; Graystock et al. 2020; Plischuk et al. 2009); both *V. ceranae* and *V. apis* are infective agents in honey bees. Additionally, *V. ceranae* infections have been confirmed infectious via microscopy in bumble bee hosts from Argentina, Uruguay, and the United Kingdom (Brown 2017). Experimental feeding experiments with *B. terrestris* have shown that bumble bees are susceptible to *V. ceranae* infection, and that workers suffer increased mortality (Graystock et al. 2013a). Bumble bees in China, Thailand, and Mexico also carried *V. ceranae*, novel strains of *Vairimorpha* that might be undescribed species, and some species of *Vairimorpha* not associated with bee hosts, but the infection status of these novel detections remains unclear (Gallot-Lavallée et al. 2016; Li et al. 2011; Sinpoo et al. 2019). A new genus and species of microsporidian, *Tubulinosema pampeana* was recently described from tissue infections in *B. atratus* hosts from Argentina, and it has also been detected in the same species in Uruguay (Plischuk et al. 2017; Plischuk et al. 2015). The only microsporidians that have been shown to cause true infections in wild bumble bees are *V. bombi, V. ceranae*, and *T. pampeana* (Brown, 2017). In addition to *A. mellifera* and *Bombus, V. ceranae* has been detected in wild European specimens of *Andrena ventralis, Heriades truncorum, Osmia bicornis*, and *O. cornuta* (Ravoet et al. 2014), with increasing evidence of active infections in *O. bicornis* (Bramke et al. 2019; Müller et al. 2019). The health impacts of *V. ceranae* on wild bee communities, especially alongside co-occurring stressors, are largely unknown.

There are a few records of ascomycetes fungi infecting bumble bees, but many members of this group are primarily saprophytic and only opportunistically pathogenic, while others are obligate pathogens of bees (Foley et al. 2014; Jensen et al. 2013; Macfarlane 1976). MacFarlane (1976) cultured a number of fungi from living and dead bumble bees, including a species of *Aspergillus*, but did not show that these fungi were capable of causing infection. In honey bees, *Aspergillus* species are the causative agents of stonebrood, a rarely observed larval malady of honey bees (Foley et al. 2014). On the whole, the *Aspergillus* are considered more saprophytic than pathogenic, but many species are capable of infecting immunocompromised hosts (both vertebrate and invertebrates) and some strains have been shown to be fully pathogenic to seemingly healthy honey bees (Foley et al. 2014; Jensen et al. 2013; Leatherdale 1970). The species *Aspergillus candidus* and *A. niger* have been recorded from bumble bee hosts, but their pathogenic roles are unclear (Macfarlane 1976; Schmid-Hempel 1999).

The 28 species of *Ascosphaera* are known as bee specialists and have been described from the nests and larvae of dozens of wild bee species, with all known cases of pathogenic *Ascosphaera* reported from larvae and causing a suite of characteristic symptoms leading to the common name chalkbrood (Wynns et al. 2013). *Ascosphaera apis* is the causative agent of chalkbrood, a larval disease of honey bees, and fungal spores are commonly found in the honey bee-sourced pollen fed to captive bumble bees (e.g., Graystock et al. 2013b; Maxfield-Taylor et al. 2015). However, recent research has reported *A. apis* infecting adult bumble bees in Oregon (United States) (Maxfield-Taylor et al. 2015). In a captive-rearing experiment, the body cavities of wild-caught queens that died prior to producing colonies were filled with vegetative and sporulating *Ascosphaera* species that the authors genetically identified as *A. apis*. Whether or not the fungus was responsible for the death of the queens or whether bumble bee larvae are also susceptible to the disease remains to be seen. However, none of the ascomycetes recorded from bumble bees have been conclusively shown to be pathogenic by satisfying Koch’s postulates, so their true status as pathogens in bumble bees is uncertain (Macfarlane 1976).

Experiments to see whether bumble bees could vector the biological control fungus *Beauveria bassiana* throughout greenhouses have shown that, at high doses, the fungus is capable of causing mortality to bees (Kapongo et al. 2007). Similar results were seen in efforts to use bumble bees as vectors of *Metarhizium anisopliae* (Smagghe et al. 2013). It is unknown how frequent infections of these fungi are in wild bumble bees, but these two fungi have been isolated from bumble bees in North America (Macfarlane 1976). Yeasts in the genus *Candida* (many now classified as *Metschnikowia*) have been cultured from bumble bees, nests, and flowers, but these are typically considered to be nectar yeasts, and likely only facultatively pathogenic to bees (Batra et al. 1973; Brysch-Herzberg 2004; Macfarlane 1976). There are other sporadic records of entomopathogenic fungi associated with bumble bees, including *Hirsutella* sp., *Acrostalagmus* sp., *Lecanicillium* (formerly *Cephalosporium* or *Verticilium*) *lecanii, Geomyces* (formerly *Chryososporium*) *pannorum*, *Parascedosporium* (formerly *Doratomyces*) *putredinis*, *Penicillium* sp., and *Isaria* (formerly *Paecilomyces*) *farinosus* (Batra et al. 1973; Goulson 2010; Macfarlane 1976; Schmid-Hempel 1999; Zimmermann 2008). An unidentified mass of hyphal growth was also described infecting the gut tissue of living adult bumble bees collected in Illinois and Oregon (United States), but the identity of this fungus remains unknown (Kissinger et al. 2011).

#### Nematodes

The nematode *Sphaerularia bombi* has a worldwide distribution with infection records in dozens of bumble bee species from North America, South America, Europe and New Zealand (Colgan et al. 2020; Goldblatt & Fell 1984; Lubbock 1861; Lundberg & Svensson 1975; Macfarlane & Griffin 1990; McCorquodale et al. 1998; Plischuk & Lange 2012; Poinar & Van Der Laan 1972). This parasite exclusively infects bumble bee queens, and upon infection, the queen is effectively sterilized. Although infected queens may live as long as uninfected queens (Macfarlane et al. 1995), they do not initiate nests upon emergence, but rather resume hibernaculum-seeking behavior (Alford 1969). Because infection with this parasite prevents queens from initiating colonies, it has the potential to impact populations severely.

Mated *S. bombi* females infect bumble bee queens as they overwinter in soil cells. They develop within the hemocoel of the host throughout the winter, maturing upon bumble bee emergence in spring. Mature, gravid females control the corpora allata of host queens, suppressing chemical signals that allow uninfected queens to mature and seek nesting sites upon emergence (Macfarlane & Griffin 1990). Each female can produce over 100,000 eggs, which are released and hatch in the hemocoel of the host queen (Macfarlane & Griffin 1990). At the third stage, juvenile nematodes burrow into the midgut of the host. These juveniles are subsequently excreted into shallow pits in the soil excavated by the infected host queen, where they will mature and wait for the next generation of overwintering queens (Poinar & Van Der Laan 1972). Because the nematodes drop into the soil to await transmission to the next generation of queens, *S. bombi* is not expected to be a pest of captive-reared bumble bees.

There are few records of mermithid parasites in bumble bee hosts, but they are geographically widespread, with records from North America, South America, Europe, and Asia (Durrer & Schmidhempel 1995; Kosaka et al. 2012; Kubo et al. 2016; MacLean 1966; Mullins et al. 2019; Plischuk et al. 2017; Rao et al. 2017; Tripodi & Strange 2018). Because the parasitic stages of mermithids are devoid of morphological characters that would allow their identification, the identity of these parasites is largely unknown. One record of a mermithid infecting a *B. impatiens* worker collected in Massachusetts (United States) was identified to the genus *Pheromermis*, but nothing is known of its life history or whether bumble bees are its primary host (Rao et al. 2017). Like *S. bombi*, these parasites require a free-living stage in the soil, so they are unlikely to present an issue in rearing facilities. Mermithids kill their hosts upon exiting the host’s body, but with so few occurrences, they are unlikely to have an impact on the population level (Tripodi & Strange 2018).

### 2) Detection, identification, and quantification

#### General techniques used to detect and quantify endosymbionts of concern

Detection of bumble bee parasites falls into two major categories: molecular methods or visual methods. Most parasite detection is destructive, requiring that bees be killed prior to examination. However, mature or transmitting infections of some parasites, including *S. bombi, Vairimorpha* spp., *Crithidia* spp., and *A. bombi*, can be visually detected in feces, a non-lethal technique (Jones & Brown 2014). For some parasites, quantification of individual parasites in feces provides an accurate estimation of the intensity of the established infection, *e.g*., for *Crithidia* (Sadd 2011). However, such a relationship has not been verified for all observable parasites detectable in the feces, and false negatives may occur during early stages of infection. In addition, low numbers of parasite transmission stages may represent false positives, where transmission stages, *e.g*., environmentally resistant extracellular *Vairimorpha* spores, are just passing through and are not from established infections. This presents an issue for any analysis where gut tissue is included and is a potential issue in both visual and molecular detection approaches. However, in closed systems, such as rearing facilities, detection of parasites and pathogens in the feces will likely represent actual infections. Although tissues of the head and mesosoma can be infected, all known parasites can be detected by examination of the tissues and hemocoel of the metasoma. Different parasites are typically detected using different techniques, but these are often complementary. Larger organisms such as nematodes are visible with light microscopy during dissection under low magnification (10–40x). This is often followed by an examination of slide-mounted tissues or homogenates at higher magnification (400x) to detect smaller organisms (*e.g*., oocytes of *Apicystis cryptica*: Schoonvaere et al. 2020). Finally, molecular methods can be used to detect, identify, and quantify parasites of all sizes from tissue extractions.

Before the development of molecular detection techniques, visual detection with light microscopy was the predominant mode of screening for internal bumble bee parasites. Light microscopy allows for the detection of parasites at 400x magnification, encompassing a broad diversity of organisms. To this day, microscopy continues to be employed in the detection and quantification of bumble bee parasites via the count of spores or cells using a hemocytometer (Fries et al. 2013). Some of the strengths of light microscopy include that it is low-cost, requires little training to employ, and most importantly, it can detect active infections through tissue pathology. However, there is a risk for false negatives as low-level or early stage infections can be missed, suggesting that traditional light microscopy may underestimate parasite prevalence (Blaker et al. 2014). False positives are also possible, especially for less-experienced researchers who are not fully aware of target parasite morphologies. In addition, many pathogens are tissue-specific, thus requiring the correct tissue to be examined for diagnosis (Schmid-Hempel 1999). However, the primary benefit of visual detection is the ability to diagnose disease and disease intensity, rather than just the presence of a potential disease-causing organism. In all cases it is preferable that known positive samples be observed under the set-up being used, to ensure accurate identification and verify the ability of the set-up to detect parasites and pathogens of interest. For example, *Crithidia* spp. require phase contrast microscopy for good visualization. However, even then, detection by observers unfamiliar with cell morphology will be aided by using fresh samples where some cells will be motile.

Polymerase Chain Reaction (PCR), developed in 1985, is the most commonly employed molecular technique for DNA amplification, and it has been used to great effect to detect parasites in both bumble bees (Blaker et al. 2014; Cordes et al. 2012; Huang et al. 2015; Koch & Strange 2012) and humans (Yang & Rothman 2004). This method uses short oligonucleotides, primers, that are designed to hybridize with known genetic regions within the genomes of targeted organisms. Samples that fail to amplify are diagnosed as negative, and samples that successfully amplify are diagnosed as positive for the targeted parasite. Including control regions that amplify bee DNA or cDNA in PCR is a common quality control measure used to guard against false negatives that can come about through poor specimen handling, nucleotide extraction or bad reactions. Positive controls should also be included in PCR to ensure viability of reactions. Similarly, the use of negative controls that contain no DNA template can help guard against false positives that usually stem from laboratory contaminants. With the development of primers for multiple targets that do not interfere with one another during thermal cycling, PCR can be multiplexed for the detection of multiple pathogens simultaneously (Huang et al. 2015; Procop 2007; Tripodi et al. 2018). One of the strengths of PCR is that it can be used to detect presence or absence of parasites at very low intensities or in small sample volumes. Quantitative PCR (qPCR) goes a step further, amplifying and detecting the target sequence simultaneously and, if properly calibrated, yielding a quantitative measure of infection intensity. For screening RNA viruses, reverse transcriptase PCR (RT-PCR) is used, which converts RNA to its complementary DNA strand (cDNA), which is then used as template in PCR (de Miranda et al. 2013). Standardized protocols for PCR-based detection of a variety of common bee pathogens have recently been released (de Miranda et al. 2021).

In a double-blind methods comparison, PCR was found to have an overall higher sensitivity for detecting human-pathogenic microsporidia compared to traditional light microscopy, though both methods proved effective (Rinder et al. 1998). Likewise, Blaker et al. (2014) found significantly higher sensitivity for detecting microsporidia in bumble bees than light microscopy detected. However, increased sensitivity is not always desirable. PCR methods do not distinguish between exposure and infection, and dead or inactivated parasites may still yield positive results. Such sensitive methods can diagnose samples as positive, regardless of the true infection status within the host, thus positive PCR results should be interpreted with this caveat in mind (Brown 2017). PCR, qPCR, and RT-PCR assays can be designed to use either species-specific or broad-range primers that can detect multiple members of a targeted taxon, depending on the desired identification level (Graystock et al. 2020; Mullins et al. 2019; Procop 2007; Yang & Rothman 2004). While broad-range primers allow for the discovery of new organisms within a targeted taxon, one of the major drawbacks of all primer-based detection techniques is that the researcher will only detect organisms or groups that are being targeted, and that detection is limited to parasites for which sequence data are available. However, post-amplification analysis of PCR products from broad-range primers through DNA sequencing can be used to identify parasites to species, generate additional data, and conduct analyses of strain differences that can be useful in understanding disease dynamics (Cameron et al. 2016).

Current advances in molecular technologies, known as next-generation sequencing (NGS) platforms, are beginning to allow for pathogen screening and sequencing through exploratory metagenomics (Gerth & Hurst 2017; Runckel et al. 2011). Exploratory work with the RNA-Seq platform recently detected a number of known bumble bee-associated organisms in two bumble bee species, as well as two undescribed viruses in *O. cornuta* (Schoonvaere et al. 2016). However, the success of these NGS techniques depends on the existence of reference databases, such as well-curated sequence deposits, knowledge of the pathology and natural history of the symbionts detected and identified, as well as the technical ability to process, analyze, and interpret the data (de Magalhães et al. 2010). As the use of these methods increases, and databases of pathogen sequences expand, NGS could provide unexplored levels of pathogen screening abilities for bumble bee research. However, despite their significant value in these regards, NGS approaches would currently be unfeasible for a rapid and high-throughput clean stock screening program, where targeted visual or molecular approaches of known parasites and pathogens of concern will be more effective.

#### Viruses

Because of their small size (typically 20–30 nm (James & Li 2012)), viruses are not visible with basic microscopy and are primarily detected through molecular methods (de Miranda et al. 2013). Using RT-PCR, specific primers can be employed to determine the presence of a virus, and the viral load can be quantified using calibrated qRT-PCR (e.g., McMahon et al. 2015). In addition, it is possible to run a multiplex RT-PCR and screen for multiple RNA viruses simultaneously (Chen et al. 2004). However, detecting the presence of a virus is not equivalent to detecting a viral infection. An advantage to the structure of many ss-RNA viruses is that it is possible to screen for their complementary strand, which, if found, indicates active replication within the host (de Miranda et al. 2013; Mazzei et al. 2014). This is not possible for DNA-based parasites.

#### Bacteria

Not all bacteria can be cultured on standard media (Přidal 2001; Shrivastava 1982) and in addition, while some can be easily viewed using standard microscopy approaches, the morphological delineation of bacterial pathogens is difficult. Therefore, molecular methods are commonly used for detection of bacteria, such as *Spiroplasma apis* and *S. melliferum* (Meeus et al. 2012). Often, culture-based and molecular methods are used in conjunction with one another in order to determine physical and chemical characteristics, experiment with inoculation and host specificity, and resolve taxonomic issues (Kwong et al. 2014; Kwong & Moran 2013; Praet et al. 2018).

#### Protozoans

The infective oocyst of neogregarines and the motile stages of trypanosomatids can be detected through microscopic examination of tissues, tissue homogenates, or fecal samples at 400x. However, these organisms have complex life cycles with cryptic vegetative growth phases that can be easily missed by microscopy, making molecular detection methods more reliable. The gross morphology of some protozoans makes their identification to broad groups rather simple, but discerning species morphologically is impossible under typical magnification. Morphological differences that separate species can be seen with scanning electron microscopy and other specialized equipment (Liu et al. 1974; Schmid-Hempel & Tognazzo 2010). *Crithidia* spp. are quite small, typically less than 10 μm long in all stages, and some stages are highly mobile and visible when alive (Schmid-Hempel & Tognazzo 2010). It is important to note that while *C. bombi* has three distinct morphological stages (amastigote: spherical form with no visible flagellum; choanomastigote: pear-like shape surrounding flagellar pocket; and promastigote: large cells with long flagellum (Logan et al. 2005; Ruiz-González & Brown 2006)), the vast majority of screening efforts via microscopy focus on the promastigote stage, potentially under-reporting infections of the other morphological stages. Spores of neogregarines are larger, 21–27 μm, and are easily visible at 400x (Liu et al. 1974). Infections can be quantified by counting oocysts in a hemocytometer (Human et al. 2013). Broad-range primers have been developed to detect trypanosomes, including *Crithidia* spp., as well as neogregarines, including *Apicystis bombi* (Meeus et al. 2010; Mullins et al. 2020; Schmid-Hempel & Tognazzo 2010). In preliminary screening, a broad-range primer may be used, then positives can be sequenced and identified (Gallot-Lavallée et al. 2016). Broadly screening and sequencing positive samples may maximize the probability of detecting potential pathogens, for groups that are likely to contain unexpected or undescribed species, such as *Crithidia*. A similar approach uses species-specific primers coupled with broad-range primers, allowing for the detection of unexpected species (Stevanovic et al. 2016; Szalanski et al. 2016; Tripodi et al. 2018).

#### Fungi

Similar to other spore-producing pathogens, visual detection of microsporidian spores at 400x is common and spore intensities can be assessed in slide-mounted tissues, homogenized gut samples, or feces smeared onto a hemocytometer (Human et al. 2013). The infective spores are the most readily distinguishable life stage of the microsporidia, as vegetative intracellular growth is cryptic and often undetectable by microscopy; however, methanol fixation and Giemsa staining can reveal these growth stages within tissue (Fries 1988). Spores of most bee-infecting microsporidia species are highly refractive in phase contrast microscopy and approximately 5 μm long. By scanning multiple visual fields at an appropriate magnification (*e.g*., 400x), repeated spore counts can be used to quantify infection levels as a concentration of spores per milliliter of homogenized tissue (Human et al. 2013) or categorized on a relative scale, such as the one used by Cordes et al. (2012) for microsporidia: low infection when <2 spores, moderate when 2–20 spores, and high infection >20 spores/ visual field (Cordes et al. 2012; Human et al. 2013). Distinguishing different species or even genera of microsporidia using light microscopy can prove difficult as the gross morphology of spores is similar across the group, although species-specific tissue pathology has been noted (Plischuk et al. 2015). PCR has higher resolution for detecting and distinguishing different microsporidia species, and species-specific primers have been developed for *V. apis, V. ceranae*, and *V. bombi* (Blaker et al. 2014; Erler et al. 2012; Graystock et al. 2020; Klee et al. 2006). Microscopy and PCR are often used in combination to maximize probability of detection while also assessing presence and intensity of sporulating infections, and are therefore complementary approaches (Blaker et al. 2014; Calhoun et al. 2021).

Entomopathogenic fungi with hyphal growth, such as chalkbrood (*Ascosphera* spp.), are uncommon in bumble bees and usually detected visually, based on the presence of hyphae in the abdominal cavity and the tissues of the alimentary tract (Kissinger et al. 2011; Macfarlane 1976; Maxfield-Taylor et al. 2015). Chalkbrood produces visible hyphae that cover the bee carcass in late stages of infection, but this pathology has only been seen in larval infections of non-*Bombus* bees (Schmid-Hempel 1999). Detection in bumble bees could include visual inspection via microscopy at low magnification (10–40x), examination of slide-mounted tissues at higher magnification (200–400x), culturing and isolation for morphological identification of reproductive structures, as well as molecular screening using broad-range or specific primer pairs (James & Skinner 2005; Macfarlane 1976; Maxfield-Taylor et al. 2015).

#### Nematodes

Due to their relatively large size, bumble bee-associated nematodes can be detected during dissections of the metasoma at low magnification (10–40x). *Sphaerularia bombi* is the most commonly encountered nematode parasite in bumble bees, although it is primarily restricted to queens (Alford 1975; Macfarlane et al. 1995). The 8–20 mm long cucumber-like inverted uterus of a mature female worm in the abdomen of the host is readily identified through dissection (Alford 1969; Plischuk & Lange 2012). Juveniles and eggs of *S. bombi* can also be detected in the feces of bees and quantified via a hemocytometer (Jones & Brown 2014). Mermithids are rarely recorded parasitizing bumble bees, but are often large (*e.g*., 46 mm in length) and easily detected during dissections at low magnification (Rao et al. 2017). The parasitic stages of mermithids lack the morphological characters to distinguish species, thus molecular characterization is recommended (Kubo et al. 2016; Tripodi & Strange 2018).

## Discussion

It is apparent that bumble bees have a considerable number of endosymbionts, which can be benign or cause sublethal or lethal pathology to their hosts. We now have techniques for detection and identification of most of these endosymbionts, yet pathologies are understudied and the impacts of detected pathogens are often unknown, particularly in diverse bumble bee host species beyond the relatively well-studied *B. impatiens* and *B. terrestris* (Cameron & Sadd 2020). It is likely that many species and strains remain to be described, and there is insufficient effort devoted to monitoring their populations in the wild. We have strong evidence that the commercial trade, both national and international, that has developed in bumble bees for use in crop pollination has facilitated the dispersal of many of these endosymbionts to non-native ranges, including around the world, and their introduction (through spillover) to wild bees. Spillover from honey bees (*Apis mellifera*) to bumble bees may also be a significant source of infection (Alger et al. 2019; Nanetti et al. 2021; Pislak Ocepek et al. 2021).

As we have pointed out, there are many examples of knowledge gaps on the topic of bumble bee endosymbionts of concern, with many recent discoveries. Some have been facilitated by the development of new analytical techniques, such as molecular screening. As additional surveys utilizing these techniques are conducted it is likely that additional species will be discovered, and that we will learn more about their geographical distributions. Just as plans are developing for national monitoring program for native bees (Woodard et al. 2020), plans should be laid for monitoring the distribution, diversity, and abundance of their parasites and pathogens.

Endosymbionts are only one category of parasites and pathogens that can affect bumble bees, and we address elsewhere the ectosymbionts that can also infect them (Evans et al. 2023), as well as the potential risk that hive products such as wax and pollen pose to wild bumble bee and other pollinators. Together, this large number of bumble bee symbionts, in the context of a large and growing national and international commercial trade in these important pollinators, demonstrates the need for regulations that will help to prevent their spread, and the associated risk to wild pollinators (Strange et al. 2023).

## Supplementary Material

Supplement1
