# Supplementary material for: Endosymbionts that threaten commercially raised and wild bumble bees (Bombus spp.)
Source: J Pollinat Ecol. Author manuscript; Available in PMC 2025 Jan 2. (PMC11694841; doi:10.26786/1920-7603(2023)713)
Supplement: Supplement1 [file NIHMS2030215-supplement-Supplement1.pdf]

DOI: 10.26786/1920-7603(2023)713

Appendix I: Symbiont list identifying potentially deleterious symbionts of concern for clean stock and commercial bumble bee rearing

The list has been sorted into two groupings based on the concern of the symbiont's presence in rearing operations: Priority/Of Concern and High Uncertainty but Potential, with select evidence presented in the main text summarized here. It is important to note that this is likely not a fully inclusive list, given current unknowns, and new symbionts of concern are likely to arise. Any clean stock program should visually monitor for problematic symptoms in individuals and colonies and verify causative agents.

Priority/Of Concern

**Symbiont**	**Evidence**
**Protozoa**	
Apicystis bombi Liu, MacFarlane, and Pengelly; Ophrocystidae	High virulence (Jones & Brown 2014; Rutrecht & Brown 2008); Potential for spillover (Graystock et al. 2013b); Possibly introduced with non-native *Bombus* (Arbetman et al. 2013)
Crithidia bombi Lipa and Triggiani; Trypanosomatidae	Virulence context dependent, but may be high (Sadd & Barribeau 2013); Easily transmitted and has been common in commercial colonies (Graystock et al. 2013b; Murray et al. 2013); High spillover potential (Colla et al. 2006)
Crithidia expoeki Schmid-Hempel and Tognazzo; Trypanosomatidae	Limited study, but assumed like *C. bombi*
**Fungi**	
Vairimorpha (Nosema) bombi Fantham and Porter; Microsporidia: Nosematidae	High virulence (Otti & Schmid-Hempel 2007; Otti & Schmid-Hempel 2008); Found in commercial distributed colonies (Graystock et al. 2013b; Murray et al. 2013)
Vairimorpha (Nosema) ceranae Fries et al.; Microsporidia: Nosematidae	Bumble bees susceptible, increases mortality (Graystock et al. 2013a)
**Viruses** (Li et al. 2011)	
Deformed wing virus (DWV); Iflaviridae	Replication in bumble bees (Levitt et al. 2013; Li et al. 2011); Pathology in commercially reared bumble bees (Genersch et al. 2006)
AKI-complex: Kashmir bee virus(KBV); *Acute Bee Paralysis Virus*(ABPV), *Kashmir Bee Virus* (KBV), *Israeli Acute Paralysis Virus* (IAPV) Dicistroviridae	Infective to bumble bees, long-lasting infectivity (Bailey & Gibbs 1964); Can be common in bumble bees (McMahon et al. 2015); Virulence may be dose- and transmission route-dependent, but can be high (Meeus et al. 2014; Niu et al. 2016; Wang et al. 2018)

High Uncertainty but Potential

**Symbiont**	**Evidence**
**Bacteria**	
Spiroplasma apis Mouches et al.; Mollicutes: Spiroplasmataceae	Disease association in honey bees and presence of bacteria in bumble bees indicates pathogenic potential, but not verified (Clark et al. 1985; Meeus et al. 2012)
Spiroplasma melliferum Clark et al.; Mollicutes: Spiroplamataceae	Associated with disease in honey bees and presence of bacteria in bumble bees indicates pathogenic potential, but not verified (Clark et al. 1985; Meeus et al. 2012)
**Viruses**	
Black queen cell virus (BQCV);<br>*Chronic Bee Paralysis Virus* (CBPV);<br>*Cloudy Wing Virus* (CWV);<br>*Sacbrood virus* (SBV);<br>*Slow Bee Paralysis Virus* (SBPV);<br>Other "honey bee" RNA viruses	Found in bumble bees, including in commercial colonies (Choi et al. 2010; McMahon et al. 2015; Peng et al. 2011; Sachman-Ruiz et al. 2015; Singh et al. 2010); Need study to understand further occurrence and virulence in bumble bees (Tehel et al. 2016)
**Fungi**	
Tubulinosema pampeana Plischuk et al.; Microsporidia; Tubulinosematidae	Detected in bumble bees, affecting adipose tissue (Plischuk et al. 2017; Plischuk et al. 2015); Currently further pathology and effects unknown.

Literature Cited

Arbetman MP, Meeus I, Morales CL, Aizen MA, Smagghe G (2013) Alien parasite hitchhikes to Patagonia on invasive bumblebee. Biological Invasions 15: 489-494. https://doi.org/10.1007/s10530-012-0311-0

Bailey L, Gibbs J (1964) Acute infection of bees with paralysis virus. Journal of Insect Pathology 6: 395-407.

Choi Y, Lee M, Hong I, Kim N, Kim H, Byeon K, Yoon H (2010) Detection of honeybee virus from bumblebee (*Bombus terrestris* L. and *Bombus ignitus*) Korean Journal of Apiculture 25: 259-266.

Clark TB, Whitcomb RF, Tully JG, Mouches C, Saillard C, Bové, J. M., Wróblewski H, Carle P, Rose DL, Henegar RB, Williamson DL (1985) *Spiroplasma melliferum*, a new species from the honeybee (*Apis mellifera*). International Journal of Systematic and Evolutionary Microbiology 35: 296-308. https://doi.org/10.1099/00207713-35-3-296.

Colla SR, Otterstatter MC, Gegear RJ, Thomson JD (2006) Plight of the bumble bee: Pathogen spillover from commercial to wild populations. Biological Conservation 129: 461-467. https://doi.org/10.1016/j.biocon.2005.11.013

Genersch E, Yue C, Fries I, de Miranda JR (2006) Detection of Deformed wing virus, a honey bee viral pathogen, in bumble bees (*Bombus terrestris* and *Bombus pascuorum*) with wing deformities. J Invertebr Pathol 91: 61-63. https://doi.org/10.1016/j.jip.2005.10.002

Graystock P, Yates K, Darvill B, Goulson D, Hughes WOH (2013a) Emerging dangers: Deadly effects of an emergent parasite in a new pollinator host. Journal of Invertebrate Pathology 114: 114-119. doi:https://doi.org/10.1016/j.jip.2013.06.005.

Graystock P, Yates K, Evison SEF, Darvill B, Goulson D, Hughes WOH (2013b) The Trojan hives: pollinator pathogens, imported and distributed in bumblebee colonies. Journal of Applied Ecology 50: 1207-1215. https://doi.org/10.1111/1365-2664.12134

Jones CM, Brown MJF (2014) Parasites and genetic diversity in an invasive bumblebee. Journal of Animal Ecology 83: 1428-1440. doi:https://doi.org/10.1111/1365-2656.12235.

Levitt AL, Singh R, Cox-Foster DL, Rajotte E, Hoover K, Ostiguy N, Holmes EC (2013) Cross-species transmission of honey bee viruses in associated arthropods. Virus Res 176: 232-240. https://doi.org/10.1016/j.virusres.2013.06.013

Li J, Peng W, Wu J, Strange JP, Boncristiani H, Chen Y (2011) Cross-species infection of Deformed Wing Virus poses a new threat to pollinator conservation. Journal of Economic Entomology 104: 732-739. https://doi.org/10.1603/EC10355

McMahon DP, Fürst MA, Caspar J, Theodorou P, Brown MJF, Paxton RJ (2015) A sting in the spit: widespread cross-infection of multiple RNA viruses across wild and managed bees. Journal of Animal Ecology 84: 615-624. https://doi.org/10.1111/1365-2656.12345

Meeus I, de Miranda JR, de Graaf DC, Wäckers F, Smagghe G (2014) Effect of oral infection with Kashmir bee virus and Israeli acute paralysis virus on bumblebee (Bombus terrestris) reproductive success. Journal of Invertebrate Pathology 121: 64-69. https://doi.org/10.1016/j.jip.2014.06.011.

Meeus I, Vercruysse V, Smagghe G (2012) Molecular detection of *Spiroplasma apis* and *Spiroplasma melliferum* in bees. Journal of Invertebrate Pathology 109: 172-174. https://doi.org/10.1016/j.jip.2011.11.006.

Murray TE, Coffey MF, Kehoe E, Horgan FG (2013) Pathogen prevalence in commercially reared bumble bees and evidence of spillover in conspecific populations. Biological Conservation 159: 269-276. http://dx.doi.org/10.1016/j.biocon.2012.10.021.

Niu J, Smagghe G, De Coninck DIM, Van Nieuwerburgh F, Deforce D, Meeus I (2016) In vivo study of Dicer-2-mediated immune response of the small interfering RNA pathway upon systemic infections of virulent and avirulent viruses in *Bombus terrestris*. Insect Biochemistry and Molecular Biology 70: 127-137. https://doi.org/10.1016/j.ibmb.2015.12.006.

Otti O, Schmid-Hempel P (2007) *Nosema bombi*: A pollinator parasite with detrimental fitness effects. Journal of Invertebrate Pathology 96: 118-124. https://doi.org/10.1016/j.jip.2007.03.016.

Otti O, Schmid-Hempel P (2008) A field experiment on the effect of *Nosema bombi* in colonies of the bumblebee *Bombus terrestris*. Ecological Entomology 33: 577-582. https://doi.org/10.1111/j.1365-2311.2008.00998.x

Peng W, Li J, Boncristiani H, Strange JP, Hamilton M, Chen Y (2011) Host range expansion of honey bee Black Queen Cell Virus in the bumble bee, *Bombus huntii*. Apidologie 42: 650-658. https://doi.org/10.1007/s13592-011-0061-5

Plischuk S, Salvarrey S, Arbulo N, Santos E, Skevington JH, Kelso S, Revainera PD, Maggi MD, Invernizzi C, Lange CE (2017) Pathogens, parasites, and parasitoids associated with bumble bees (*Bombus* spp.) from Uruguay. Apidologie 48: 298-310. https://doi.org/10.1007/s13592-016-0474-2

Plischuk S, Sanscrainte ND, Becnel JJ, Estep AS, Lange CE (2015) *Tubulinosema pampeana* sp. n. (Microsporidia, Tubulinosematidae), a pathogen of the South American bumble bee *Bombus atratus*. Journal of Invertebrate Pathology 126: 31-42. doi:https://doi.org/10.1016/j.jip.2015.01.006.

Rutrecht ST, Brown MJF (2008) The life-history impact and implications of multiple parasites for bumble bee queens. International Journal for Parasitology 38: 799-808. https://doi.org/10.1016/j.ijpara.2007.11.004.

Sachman-Ruiz B, Narváez-Padilla V, Reynaud E (2015) Commercial *Bombus impatiens* as reservoirs of emerging infectious diseases in central México. Biological Invasions 17: 2043-2053. https://doi.org/10.1007/s10530-015-0859-6

Sadd BM, Barribeau SM (2013) Heterogeneity in infection outcome: lessons from a bumblebee-trypanosome system. Parasite Immunology 35: 339-349. doi:https://doi.org/10.1111/pim.12043.

Singh R, Levitt AL, Rajotte EG, Holmes EC, Ostiguy N, vanEngelsdorp D, Lipkin WI, dePamphilis CW, Toth AL, Cox-Foster DL (2010) RNA viruses in Hymenopteran pollinators: Evidence of inter-taxa virus transmission via pollen and potential impact on non-*Apis* Hymenopteran species. PLOS ONE 5: e14357. https://doi.org/10.1371/journal.pone.0014357

Tehel A, Brown MJF, Paxton RJ (2016) Impact of managed honey bee viruses on wild bees. Current Opinion in Virology 19: 16-22. https://doi.org/10.1016/j.coviro.2016.06.006.

Wang H, Meeus I, Piot N, Smagghe G (2018) Systemic Israeli acute paralysis virus (IAPV) infection in bumblebees (*Bombus terrestris*) through feeding and injection. Journal of Invertebrate Pathology 151: 158-164. https://doi.org/10.1016/j.jip.2017.11.015
